# Successful Multimodal Treatment of Intracranial Growing Teratoma Syndrome with Malignant Features

**DOI:** 10.3390/curroncol31040138

**Published:** 2024-03-29

**Authors:** Daiken Satake, Manabu Natsumeda, Kaishi Satomi, Mari Tada, Taro Sato, Noritaka Okubo, Keita Kawabe, Haruhiko Takahashi, Yoshihiro Tsukamoto, Masayasu Okada, Masakazu Sano, Haruko Iwabuchi, Nao Shibata, Masaru Imamura, Chihaya Imai, Hirokazu Takami, Koichi Ichimura, Ryo Nishikawa, Hajime Umezu, Akiyoshi Kakita, Makoto Oishi

**Affiliations:** 1 Department of Neurosurgery, Brain Research Institute, Niigata University, Niigata 951-8585, Japan; s.d.341211@gmail.com (D.S.); sppw57t9@gmail.com (T.S.); nokubo0306@yahoo.co.jp (N.O.); kawabe0925@gmail.com (K.K.); haruhiko.takahashi@icloud.com (H.T.); y-tsukamoto@bri.niigata-u.ac.jp (Y.T.); masayasu_okd@bri.niigata-u.ac.jp (M.O.); masa2188@icloud.com (M.S.); mac.oishi@bri.niigata-u.ac.jp (M.O.); 2 Advanced Treatment of Neurological Diseases Branch, Brain Research Institute, Niigata University, Niigata 951-8585, Japan; 3 Department of Pathology, Kyorin University Faculty of Medicine, Tokyo 181-8611, Japan; kaishi-satomi@ks.kyorin-u.ac.jp; 4 Department of Pathology, Brain Research Institute, Niigata University, Niigata 951-8585, Japan; tadamari@bri.niigata-u.ac.jp (M.T.); kakita@bri.niigata-u.ac.jp (A.K.); 5 Department of Pediatrics, Niigata University Medical and Dental Hospital, Niigata 951-8520, Japan; halgon77@med.niigata-u.ac.jp (H.I.); shibata-nao@med.niigata-u.ac.jp (N.S.); chihaya@med.u-toyama.ac.jp (C.I.); 6 Department of Pediatrics, Toyama University, Toyama 930-0194, Japan; 7 Department of Neurosurgery, The University of Tokyo Hospital, Tokyo 113-8655, Japan; takamih-nsu@h.u-tokyo.ac.jp; 8 Department of Brain Disease Translational Research, Juntendo University Graduate School of Medicine, Tokyo 113-8421, Japan; k.ichimura.uk@juntendo.ac.jp; 9 Department of Neurosurgery/Neuro-Oncology, Saitama Medical University International Medical Center, Saitama 350-1298, Japan; rnishika@saitama-med.ac.jp; 10 Division of Pathology, Niigata University Medical and Dental Hospital, Niigata University, Niigata 951-8520, Japan; umezu@med.niigata-u.ac.jp

**Keywords:** growing teratoma syndrome, multimodal treatment, methylation classifier, copy number variation analysis

## Abstract

Molecular analysis of the growing teratoma syndrome has not been extensively studied. Here, we report a 14-year-old boy with a growing mass during treatment for a mixed germ cell tumor of the pineal region. Tumor markers were negative; thus, growing teratoma syndrome was suspected. A radical resection via the occipital transtentorial approach was performed, and histopathological examination revealed a teratoma with malignant features. Methylation classifier analysis confirmed the diagnosis of teratoma, and *DMRT1* loss and 12p gain were identified by copy number variation analysis, potentially elucidating the cause of growth and malignant transformation of the teratoma. The patient remains in remission after intense chemoradiation treatment as a high-risk germ cell tumor.

## 1. Introduction

Growing teratoma syndrome is a rare condition in which the paradoxical growth of a mature teratoma occurs during treatment for a mixed malignant germ cell tumor, despite normalization of tumor markers. It was first described in 1982 by Logothetis et al. [1]. Molecular studies of growing teratoma syndrome have not been extensively reported, and its pathophysiology remains largely obscure. We describe a 14-year-old boy with a growing mass during treatment for a mixed germ cell tumor of the pineal region. A radical resection was performed, and histopathological examination revealed a teratoma with malignant features. *DMRT1* loss and 12p gain were identified by copy number variation analysis, potentially elucidating the cause of the formation and malignant phenotype of the teratoma. Intensive chemoradiation treatment was performed because morphological and molecular evidence of malignancy was observed.

## 2. Case Presentation

**Figure 1:**
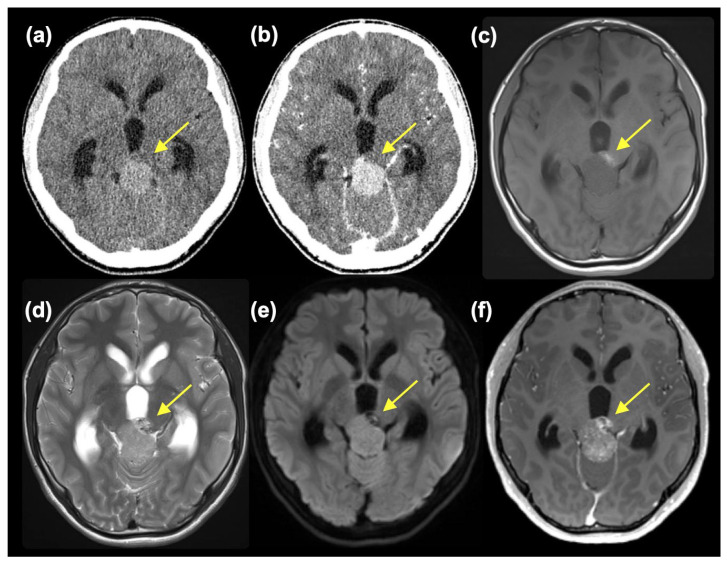
Pre-contrast (**a**) and post-contrast CT (**b**) and T1WI (**c**), T2WI (**d**), DWI (**e**), and post-contrast MRI (**f**) showing two components (small component indicated with yellow arrow).

A 14-year-old boy without a notable history presented with worsening headaches, blurry vision, and vomiting. An MR scan taken at a neurosurgery clinic revealed a mass in the pineal region and accompanying obstructive hydrocephalus. He was referred to the Department of Neurosurgery, Niigata University, for further evaluation and treatment. At the initial presentation, he had a severe headache, and mild impairment of consciousness was observed (Glasgow Coma Scale 15 E4V5M6, but with mild disorientation). Extraocular movement was objectively normal, but the patient complained of blurry vision. No other neurological symptoms were observed. A head CT scan showed a slightly high-dense tumor mass, approximately 3.3 cm in diameter, at the pineal region with marked enlargement of bilateral lateral and third ventricles (Figure 1a). The tumor showed homogeneous enhancement, except for a small area at the left anterior part of the tumor, which was isodense on pre-contrast CT and did not enhance (Figure 1b). MR images also depicted tumor regions with different intensities. The main part of the tumor was isointense on T1-weighted images (T1WI) and slightly hyperintense on T2-weighted images (T2WI) and diffusion-weighted images (DWI) with moderate enhancement. In retrospect, these intensities were thought to reflect calcification, although plain CT was not highly dense. On the other hand, the left anterior portion of the tumor was hyperintense on T1WI, mixed hyper- and hypointense on T2WI, hypointense on DWI, and showed heterogeneous enhancement (Figure 1c–f). A mixed type of germ cell tumor was suspected. However, at presentation, serum tumor markers were negative (AFP 5 ng/mL and β-HCG 6 mIU/mL).

**Figure 2:**
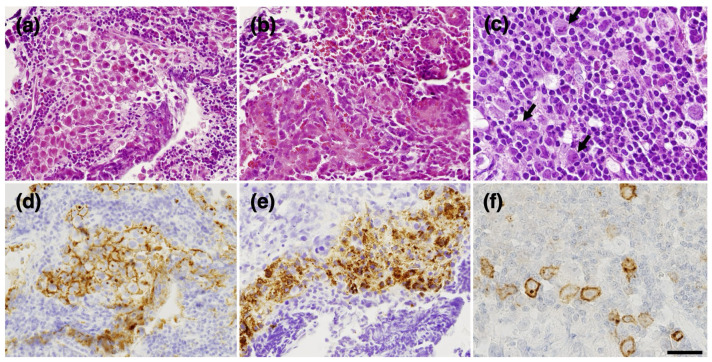
Pathological findings of the initial surgery. The germinoma component showing a classic two-cell pattern (**a**) with PLAP-positive tumor cells (**d**) and small lymphoid cells. The AFP-positive component forms a thick epithelial cord (**b**,**e**) with a small glandular structure (inset in (**e**)). Small calcification deposits are observed in this region (inset in (**b**)). CD30-positive component (**c**,**f**). CD-30 labels large cells with severe nuclear atypia (arrows in (**c**)). Immunohistochemistry (**d**–**f**). (**d**), PLAP; (**e**), AFP; (**f**), CD30. Bar = 50 μm for (**a**,**b**,**d**,**e**), 25 μm for (**c**,**f**), and 35 μm for insets in (**b**,**e**).

An endoscopic biopsy and a third ventriculostomy were performed using a flexible endoscope. Both components of the tumor were sampled. The former was whitish and relatively soft; the latter component was obviously harder. The first component was histologically a germinoma component (Figure 2a), consisting of large, round tumor cells staining for placental alkaline phosphatase (PLAP) (Figure 2d) and mixed with small lymphocytes, and an embryonal carcinoma-like component (Figure 2b,c), which showed epithelial features and calcification (Figure 2b), as well as immunoreactivity for cytokeratin and partly for AFP (Figure 2e) and CD30 (Figure 2f). Intraoperatively, cerebrospinal fluid (CSF) was obtained from inside the ventricle. CSF PLAP (1280 pg/mL) and β-HCG (15 mIU/mL) were elevated, but surprisingly, AFP was not elevated (5 ng/mL).

**Figure 3:**
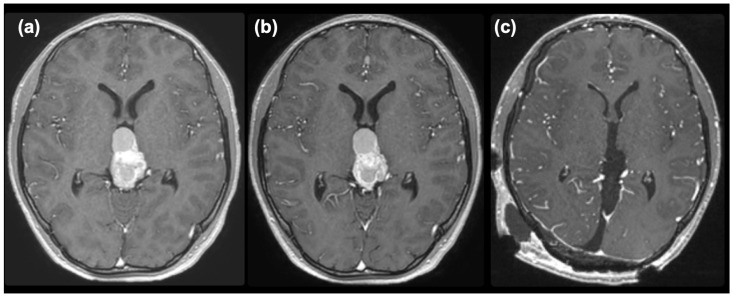
Post-contrast MR images taken after one course (**a**), two courses (**b**) of chemotherapy, and after removal (**c**).

The mixed germ cell tumor was determined to be of intermediate risk according to the Matsutani classification [2,3], and a chemotherapeutic regimen followed by irradiation was planned. First, a regimen of carboplatin (450 mg/m^2^) and etoposide (150 mg/m^2^ × 3 days) was commenced. An elevation of serum AFP to 81 ng/mL was noted after the commencement of chemotherapy, and enlargement of the tumor and cystic component was observed on MRI (Figure 3a). After two courses of chemotherapy, serum AFP had normalized. However, the size of the tumor did not decrease (Figure 3b), so we decided to remove the tumor under the suspicion of growing teratoma syndrome. The pineal tumor was completely removed via the occipital transtentorial approach [4] (Figure 3c). The recurrent tumor was very hard and was resected en-bloc after the puncture of the cyst.

**Figure 4:**
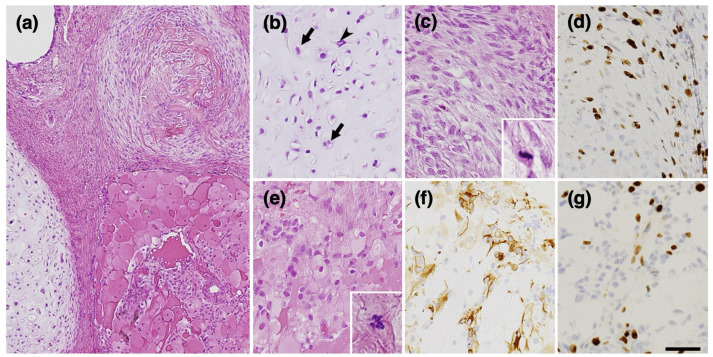
Pathologic findings of the second surgery. Teratomatous tumor with cartilage, bone, fibroblasts, and mesenchymal tissue (**a**). Cartilage tissue with relatively high cellularity containing chondrocytes with nuclear atypia (arrowhead) and frequent double nuclei (arrows). (**b**) Immature mesenchymal tissue (**c**) with a high MIB-1 labeling index of 30% (**d**). Plump eosinophilic large cells (**e**) with GFAP-positive small cells (**f**). The MIB-1 labeling index in this area is 13% (**g**). Immunohistochemistry ((**d**,**f**,**g**)). (**d**,**g**), MIB-1; (**f**), GFAP. Bar = 120 µm for (**a**), 50 µm for (**b**–**g**), and 10 µm for insets in (**c**,**e**).

Pathological examination revealed a teratomatous tumor with components of cartilage, bone, and fibroblasts (Figure 4a). However, upon closer examination, striking malignant features such as cartilage consisting of chondrocytes with nuclear atypia and frequent double nuclei (Figure 4b) and immature mesenchymal tissue with ossification (Figure 4a,c) were revealed. Although no embryonic neuroepithelial element was observed, there were GFAP-positive small cells with several mitotic figures (Figure 4e), probably representing neuroectodermal elements. Also, MIB-1 staining was diffusely elevated (Figure 4d,g), which is not consistent with the traditional definition of growing teratoma syndrome.

After thorough deliberation in a multi-disciplinary group, including pathologists, pediatric oncologists, radiation oncologists, and neurosurgeons, we decided to treat the patient as a high-risk patient further, considering that a teratoma with malignant features had enlarged even after treatment as an intermediate-risk mixed germ cell tumor. The patient subsequently received craniospinal irradiation (CSI) at 30.6 Gy (17 fractions (fr)) and boost (23.4 Gy) to the tumor removal cavity, followed by six cycles of ifosfamide (900 mg/m^2^ × 5 days), cisplatin (20 mg/m^2^ × 5 days), and etoposide (60 mg/m^2^ × 5 days) (ICE) with autologous stem cell transplantation. The patient has remained relapse-free for over three years since the initial presentation. 

**Figure 5:**
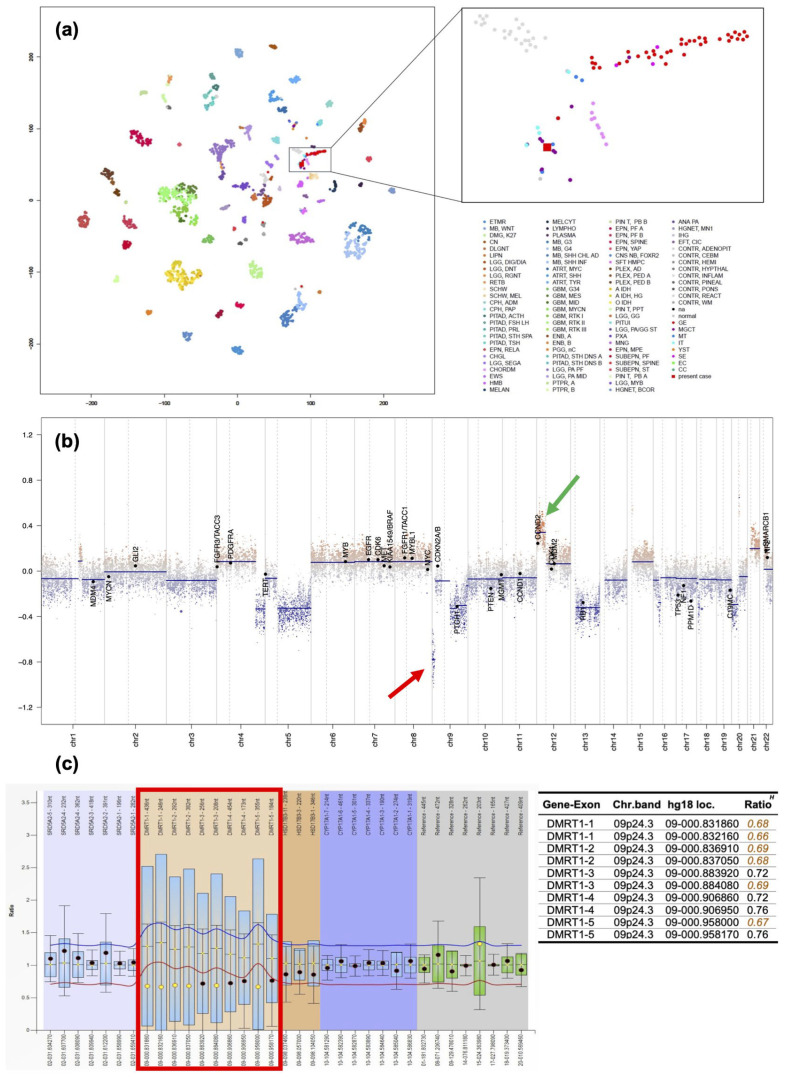
(**a**) tSNE analysis of a recurrent tumor in the present case (**b**) Copy number variation analysis revealing *DMRT1* loss (red arrow) at chromosome 9 and 12p gain (green arrow). (**c**) MLPA analysis confirmed *DMRT1* loss (red frame).

To confirm the pathological diagnosis, methylation classifier analysis ((the DKFZ classifier, developed and supplied by the German Cancer Research Center (Deutsches Krebsforschungszentrum, DKFZ) ver. 12.8 (https://www.molecularneuropathology.org) (accessed on 11 February 2024)) was performed after approval by the Institutional Review Board of Niigata University (#G2022-0012) and obtaining written consent from the patient and family. The tumor matched the methylation class of teratoma with a high calibrated score of 0.99. Furthermore, t-distributed stochastic neighborhood embedding (t-SNE) analysis using relevant data provided by Capper et al. [5] and the Intracranial Germ Cell Tumor Genome Analysis (iGCT) Consortium [6] revealed that, interestingly, this tumor fell in the vicinity of mixed germ cell tumors (MGCT), mature teratomas (MT), and immature teratomas (IT) (Figure 5a). Furthermore, analysis of copy number variation analysis showed *DMRT1* loss at chromosome 9, possibly the trigger of teratoma formation, and 12p gain, which we have previously reported to be a marker of aggressive non-germinomatous germ cell tumors (NGGCTs) of the central nervous system (CNS) and seen in approximately 30% of cases [6] (Figure 5b). However, 3p25.3 gain, a recently discovered independent predictor of poor prognosis in testicular, mediastinal [7], and CNS germ cell tumors [8], was not identified in the present case. We verified *DMRT1* loss by MLPA analysis (Figure 5c) using the SALSA MLPA P334-A3 Gonadal kit (MARC Holland, Amsterdam, The Netherlands).

## 3. Discussion

Growing teratoma syndrome is a condition in which mature teratoma with negative tumor markers arises at the site of a treated malignant germ cell tumor. It was first described in 1982 by Logothetis et al. [1]. Several studies have documented growing teratoma syndrome, but mostly in adults. To date, only 98 pediatric growing teratoma syndrome patients, of which 53 (54%) were intracranial, have been reported [9]. Growing teratoma syndrome is considered rare, but a 5% frequency of intracranial growing teratoma syndrome has been reported [10]. Classically, growing teratoma syndrome is thought to be a histologically benign mature teratoma, and growth is mainly attributed to the enlargement of the multicystic component of mature teratoma [11].

The genetic background of growing teratoma syndrome has yet to be extensively studied. A cancer predisposition syndrome caused by a *PTEN* gene variant was reported in a 2-year-old girl with a malignant ovarian germ cell tumor, whose treatment was complicated by the growing teratoma syndrome [12]. In the present case, copy number variation analysis revealed *DMRT1* loss and 12p gain. DMRT1, expressed in primordial germ cells, is known to drive the reprogramming and propagation of tumor cells, which have the capacity to induce pluripotent stem cells, leading to the development of cancer that resembles human germ cell tumors [13]. *Dmrt1* acts as a dose-sensitive tumor suppressor gene in 129Sv mice, and loss of *Dmrt1* has been shown to cause a high incidence of testicular teratomas in mice of the 129Sv strain [14]. We can speculate that in the present case, *DMRT1* loss caused differentiation into various tissue types, such as cartilage and bone, leading to the formation of teratoma. Interestingly, *DMRT1* loss was not observed in any of the 83 cases with completed copy number analyses in the iGCT Consortium cohort, suggesting that it is not a common event. It remains to be seen whether *DMRT1* loss contributes to teratomagenesis in growing teratoma syndrome, and matched pair analysis of copy number alterations between primary and recurrent CNS GCTs will be vital. Unfortunately, due to the small sample size of the tumor during the first surgery, we were unable to perform methylation classifiers and copy number variation analyses. *DMRT1* loss was verified in the recurrent tumor by MLPA (Figure 5c). 

12p gain has been implicated as a genetic hallmark of non-germ cell neoplasia in situ (non-GCNIS) in testicular germ cell tumors (TGCTs) [15,16,17,18] and is considered to be associated with the acquisition of invasiveness in these tumors [19]. We have previously shown that in CNS GCTs, 12p gain is mutually exclusive with *KIT* mutations, is abundant in NGGCTs, and is associated with significantly shorter overall and progression-free survival [6]. Although further functional confirmation is needed, the results of copy number variation analysis provide clues as to the causes of the formation and malignant phenotype of growing teratoma.

A recent report longitudinally studied DNA methylation, microRNA expression, and secretome in two growing teratoma syndrome patients. They found the presence of pluripotency- and yolk-sac tumor-associated genes preceding the formation of yolk-sac tumor or somatic-type malignancy, suggesting that growing teratoma syndrome is a continuously growing teratoma that may harbor occult non-seminomatous components considerably reduced during chemotherapy but with the potential to outgrow over time [20]. These new data have potential clinical implications, and they stress the importance of serial assessment of biological markers in the growing teratoma syndrome.

The classical definition of growing teratoma syndrome involves the histopathological confirmation of a benign, mature teratoma. In the present case, a teratoma with apparent malignant features was observed, and intense chemoradiation treatment was applied. In such cases, chemoradiation treatment may be necessary to eradicate the tumor, whereas in classically growing teratoma cases, only prompt and complete surgical removal is necessary. Careful pathological examination is vital, and molecular analysis can be a helpful adjunct in understanding the biology of these recurrent tumors.

In the present case, we obtained tissue samples from seven different parts of the tumor, including the two radiologically distinct areas. However, we were unable to find evidence of teratoma components in the initial tumor. A more aggressive surgical approach may be considered when tumor markers are positive. However, in the present case, serum AFP and HCG were initially negative. In light of the AFP-positive embryonal carcinoma-like component obtained during the initial biopsy, we decided to treat the patient as intermediate risk. There may be debate as to whether we should have initially treated the patient more intensively, but there is always a difficult clinical judgment when a discrepancy between tumor markers and pathology exists, as in the present case.

In treating the relapsed tumor, we determined that the relapsed tumor was a teratoma with malignant features. Thus, we decided to treat it with intensive chemoradiation and succeeded in eradicating the tumor. Close monitoring of potential relapse with serial MR imaging and analysis of tumor markers are being performed.

In conclusion, much remains unknown regarding the pathophysiology of growing teratoma syndrome. The presence of growing teratomas with malignant features, such as in the case presented, can further confound this rare condition. Performing molecular analysis of individual cases may help elucidate the pathophysiology of this complex syndrome.

## Data Availability

The data presented in this study are available upon reasonable request from the corresponding author.
